# Supplementary material for: The genome sequence of an ichneumonid wasp, Hyposoter dolosus (Gravenhorst, 1829)
Source: Wellcome Open Res. 2024 Sep 3;9:499. [Version 1] doi: 10.12688/wellcomeopenres.22895.1 (PMC11443231; doi:10.12688/wellcomeopenres.22895.1)
Supplement: Peer review history

# Reviewer report: Reviewer response for version 1

**DOI:** 10.21956/wellcomeopenres.25211.r99042

**Published:** 2024-09-30

**Authors:** Bernardo F Santos

**Affiliations:** 1 Institut de Systématique, Evolution, Biodiversité (ISYEB), rue Cuvier CP50, Paris Cedex, France

This is yet another very invaluable contribution from the DToL project. It follows closely the format of previous genome releases. The methods are well described and the introduction and results are elegantly written. Give its descriptive, straightforward nature, I don't think any changes are necessary and therefore recommend indexing as it stands.

Are sufficient details of methods and materials provided to allow replication by others?

Yes

Is the rationale for creating the dataset(s) clearly described?

Yes

Are the datasets clearly presented in a useable and accessible format?

Yes

Are the protocols appropriate and is the work technically sound?

Yes

Reviewer Expertise:

Systematics, phylogenomics and evolution of parasitoid wasps.

I confirm that I have read this submission and believe that I have an appropriate level of expertise to confirm that it is of an acceptable scientific standard.

# Reviewer report: Reviewer response for version 1

**DOI:** 10.21956/wellcomeopenres.25211.r99041

**Published:** 2024-09-13

**Authors:** Zachary Lahey

**Affiliations:** 1 U. S. Vegetable Laboratory, USDA-ARS, Charleston, South Carolina, USA

The purpose of this manuscript is to describe the genome sequence of Hyposoter dolosus, a species of parasitic Hymenoptera in the family Ichneumonidae. Ichneumonoids are an incredibly diverse group of parasitoids and having a chromosome scale genome assembly will be a boon to future research on the evolution of the family. The manuscript is well written and easy to follow. The methodology appears sound, and the data used to generate the genome assembly are freely available. I see no hurdles to publication of this article. I made minor corrections/suggestions to the PDF version of the article which I will email directly to the author should no option be available to upload it here.

 The only component of this analysis that stands out as odd is the length of the mitochondrial genome which is approximately 10 kb larger than the typical length of a metazoan mitochondrion. I downloaded the mitochondrial genome using the GenBank accession number reported in this publication and ran the genome through MITOS2. No major anomalies were detected. One tRNA is missing (trnF) and the rrnL is duplicated (this may be an annotation error). The "control" region is very large and runs from position 1790 to 13377. The control region usually is not that large. This should be explored further and could potentially be a standalone publication on its own. Perhaps a sentence should be added that compares the length of the mtgenome in this species to others in Ichneumonoidea.

 Overall, I think it's a tidy article and worthy of indexing.

 Zach Lahey

Are sufficient details of methods and materials provided to allow replication by others?

Yes

Is the rationale for creating the dataset(s) clearly described?

Yes

Are the datasets clearly presented in a useable and accessible format?

Yes

Are the protocols appropriate and is the work technically sound?

Yes

Reviewer Expertise:

Systematics, taxonomy, and evolution of parasitic Hymenoptera; genomics; biological control of sternorrhynchous Hemiptera

I confirm that I have read this submission and believe that I have an appropriate level of expertise to confirm that it is of an acceptable scientific standard.
